# Manifestation of Congenital CMV-Related Hearing Loss in Cohort Followed at Ear, Nose, and Throat Clinic

**DOI:** 10.3390/audiolres15050139

**Published:** 2025-10-15

**Authors:** Hajime Koyama, Akinori Kashio, Teru Kamogashira, Aki Sakata, Shinji Urata, Anjin Mori, Kenji Kondo

**Affiliations:** Department of Otorhinolaryngology, Head and Neck Surgery, The University of Tokyo, Tokyo 113-8655, Japan

**Keywords:** cytomegalovirus-associated hearing loss, progression, intellectual disability

## Abstract

**Background/Objectives:** Cytomegalovirus (CMV)-associated hearing loss is common in non-genetic congenital hearing loss. Despite this high prevalence, a wide range of clinical characteristics exists, and the pattern of hearing loss remains unknown. This study aims to describe the clinical manifestations in children with CMV-associated hearing loss and to clarify the timing of hearing level change and the degree of hearing level fluctuation. **Methods:** A total of 54 patients with hearing loss due to congenital CMV infection were included. Hearing loss type (congenital or later onset), hearing loss laterality (unilateral or bilateral), severity at first and last visit, hearing progression and timing, and the difference between patients with intellectual disability and without intellectual disability were assessed. **Results:** The number of patients with congenital hearing loss and later onset hearing loss were 19 patients and 13 patients, respectively. Seventy-four percent (14/19) of the congenital hearing loss patients and 62% (8/13) of the later onset hearing loss patients eventually progressed to severe to profound hearing loss bilaterally. Progression occurred in less than 1 year (9 cases), between 1 and 3 years (7 cases), between 3 and 7 years (4 cases), or more than 8 years (1 case). Multiple progression events occurred in 11 cases. **Conclusions:** Sixty-one percent of patients had progression of hearing loss. Several cases experienced progression over more than one year and showed multiple progression events. CMV patients without intellectual disability tended to suffer later onset hearing loss. Sixty-nine percent of the patients eventually progressed to bilateral severe to profound hearing loss, which means that continuous long-term follow-up is required.

## 1. Introduction

Cytomegalovirus (CMV) is a ubiquitous herpesvirus that infects people of all ages and is widely prevalent across the world. When CMV infection occurs in utero, it can lead to a variety of congenital abnormalities, including hepatomegaly, microcephaly, and neurological impairments such as developmental delay and motor dysfunction. Hearing loss is recognized as one of the most frequent and impactful complications. In Japan, for example, CMV-related hearing loss accounts for approximately 17% of all cases of congenital hearing loss in children, highlighting its public health significance [1].

CMV-associated hearing loss is now known to be the most common cause of non-genetic sensorineural hearing loss in children [2], but the clinical presentations vary widely among affected individuals. Hearing loss severity can range from mild to profound and can be present in one ear (unilateral) or both ears (bilateral). Additionally, some children exhibit fluctuating patterns experience a progressive decline in hearing over time [3] or have hearing loss that is not present at birth but appears later in life, a condition known as later onset hearing loss [4,5]. This variability makes it difficult for clinicians to establish standardized management strategies for affected patients.

Some papers have attempted to describe this variety of clinical manifestations [6]. However, much remains unknown. In particular, it is unclear when hearing fluctuation occurs, what degree of hearing level is reached, and what other clinical symptoms are associated with the hearing loss pattern.

In this paper, we aim to describe the clinical manifestation in children with CMV-associated hearing loss and clarify the timing of changes in hearing levels, the degree of hearing level after fluctuation, and the association with other clinical symptoms.

## 2. Materials and Methods

### 2.1. Study Design

A retrospective medical chart review was performed at a single tertiary center. A total of 54 patients visited our department due to hearing loss caused by congenital cytomegalovirus (cCMV) infection between January 2014 and December 2023. Diagnosis was made through CMV DNA detection in urine within three weeks after birth or in the dry umbilical cord by polymerase chain reaction.

Patients were diagnosed with CMV in various clinical settings and referred to our department via multiple pathways. Some were identified in pediatric departments after suspected hearing loss was detected by tests such as auditory brainstem response and subsequently referred for further audiological evaluation. Others were diagnosed with hearing loss at otorhinolaryngology facilities but referred for CMV assessment because those facilities could not diagnose CMV using a dried umbilical cord. In some cases, CMV-associated hearing loss had already been diagnosed, and the patients were referred for cochlear implantation. Hearing loss was also identified in patients evaluated for delayed language development or poor auditory responsiveness in pediatric or ENT clinics, with CMV infection subsequently confirmed.

The study was conducted in compliance with the principles of the Declaration of Helsinki. Due to the retrospective nature of the study, informed consent was not required. This study was approved by the Research Ethics Committee of the University of Tokyo Faculty of Medicine (approval number 2487).

### 2.2. Assessment Variables and Follow-Up Periods

The following were examined: age at first visit; hearing loss type (congenital or later onset); hearing loss laterality (unilateral or bilateral); hearing loss severity at first and latest visits; progression of hearing loss and timing of progression; and presence of intellectual disability. The age of referral was set as the age of the first visit. A newborn hearing screening test was performed in maternity hospitals using automated auditory brainstem response or distortion product otoacoustic emissions. Hearing level was assessed using the following audiometric function tests.

Hearing assessment follow-ups were performed every 3 months after the first visit for at least one year after the initial referral or until they reached 3 years old, and the interval was extended to 6 months when the need for clinical review was less needed. We recommend that all patients have a hearing check-up twice a year. However, some patients have dropped out.

### 2.3. Audiometric Function Tests

#### 2.3.1. Auditory Brainstem Responses (ABRs)

ABRs were recorded using the Eclipse system (Interacoustics, Middelfart, Denmark) with an electrode montage of vertex (CZ) to the ipsilateral (stimulated) ear lobe and ground to forehead (Fz). Narrow-band CE chirps^®^ were used for the stimulation. Hearing level was determined as the threshold at which wave V disappeared.

#### 2.3.2. Conditioned Orientation Reflex Responses (CORs)

CORs were performed in a soundproof room with a variety of toys. The children were conditioned to turn their heads toward the source of the sound by showing them the sound and the toys. Their hearing level was determined at the threshold at which they responded at 500, 1000, and 2000 Hz. The average hearing level was calculated using the following formula: (500 Hz + 1000 Hz × 2 + 2000 Hz) ÷ 4.

#### 2.3.3. Pure Tone Audiometries (PTAs) Including Play Audiometries

PTAs were recorded using clinical audiometers (AA-H1, Rion, Tokyo, Japan). Air conduction threshold frequencies were measured at 125, 250, 500, 1000, 2000, 4000, and 8000 Hz. For young children who were unable to press a button in response, we asked them to perform a game action in response to a sound stimulus (play audiometry). The average hearing level was calculated using the following formula: (500 Hz + 1000 Hz × 2 + 2000 Hz) ÷ 4.

### 2.4. Definitions

#### 2.4.1. Hearing Loss Laterality

Unilateral hearing loss was determined when the patient’s hearing level was above 25 dB on one side and below 25 dB on the other side. Bilateral hearing loss was determined when the patient’s hearing level was above 25 dB on both sides.

#### 2.4.2. Hearing Loss Severity

Hearing levels were classified into five categories: normal, mild, moderate, severe, and profound. A normal hearing level was defined as <25 dB, mild hearing loss as between ≥25 dB and <40 dB, moderate hearing loss as between ≥40 dB and <70 dB, severe hearing loss as between ≥70 dB and 90 dB, and profound hearing loss as ≥90 dB.

#### 2.4.3. Hearing Loss Progression

A patient was diagnosed with hearing loss progression when their hearing loss severity changed. The timing of hearing loss progression was determined from when the patient’s hearing loss progression was first evaluated.

#### 2.4.4. Symptomatic cCMV Infection and cCMV Infection with Isolated Hearing Loss

Symptomatic cCMV infection was defined as the presence of one or more cCMV-related manifestations other than hearing loss, including intellectual disability, developmental delay, pancytopenia, epilepsy, or visual impairment. cCMV infection with isolated hearing loss was defined as the presence of hearing loss as the sole manifestation.

#### 2.4.5. Congenital Hearing Loss and Later Onset Hearing Loss

We defined congenital hearing loss as a condition in which patients were suspected of having hearing loss in the newborn hearing screening and were confirmed to have hearing loss at tertiary centers, including our institution. Patients suspected of having hearing loss during the newborn hearing screening but who were later confirmed to have no hearing loss, such as those with otitis media with effusion that improved later, were excluded from the congenital hearing loss group. Later onset hearing loss was defined as a condition in which patients passed the newborn hearing screening bilaterally but were later confirmed to have hearing loss.

### 2.5. Statistics

Fisher’s exact test was used to assess the difference between the two groups. We set statistical significance to *p* < 0.05.

## 3. Results

The average age of the 54 patients at the first visit was 2 years and 2 months old (range: 1 month to 17 years old). The average age of the 54 patients at the last visit was 6 years and 6 months old (range: 8 months to 23 years old), and the average follow-up period was 4 years and 3 months (range: 8 months to 23 years). Of those patients, 24 had symptomatic cCMV infection and 30 had cCMV infection with isolated hearing loss, and 36 patients suffered sensorineural hearing loss. There were 19 cases of congenital sensorineural hearing loss and 13 cases of later onset sensorineural hearing loss. The average age at which later onset hearing loss patients experienced the onset of hearing loss was 2 years and 5 months (range: 3 months to 6 years). All the later onset hearing loss patients were referred to our institution without follow-up in the referral hospital. Of the 22 patients with no congenital or later onset sensorineural hearing loss, four had an unknown onset of sensorineural hearing loss, nine had otitis media effusion, two had conductive hearing loss without otitis media effusion, and seven had a normal hearing threshold. A total of 11 patients (8 congenital hearing loss patients, 2 later onset hearing loss patients, and 1 unknown onset hearing loss patients) received valganciclovir (Table 1). The treatment strategy, including patient selection, timing of treatment, and duration of treatment, depended on each pediatric department and was inconsistent. There was no statistical difference in the prevention of hearing loss progression between patients who received the antiviral drug and those who did not (*p* = 0.14)

### 3.1. Hearing Loss at the First Visit Among Patients with Congenital Hearing Loss

**Figure 1:**
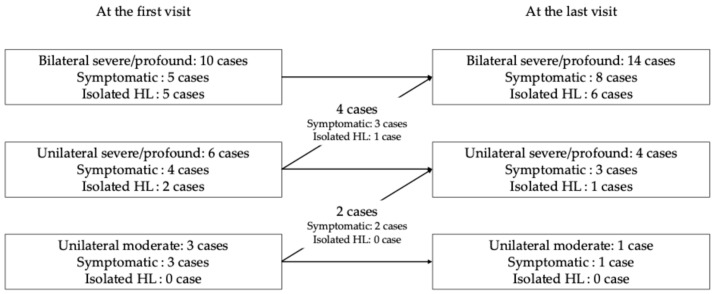
The severity and laterality of hearing loss at the first visit among congenital hearing loss patients at the first visit and the last visit. All ten bilateral severe/profound cases and four unilateral severe/profound cases showed bilateral severe/profound hearing loss (14 = 10 + 4). Two (2 = 6 − 4) unilateral severe/profound cases and two unilateral moderate cases showed unilateral severe/profound hearing loss (4 = 2 + 2). Only one (1 = 3 − 2) unilateral moderate case showed unilateral severe and moderate hearing loss. HL; hearing loss.

Figure 1 shows the severity and laterality of hearing loss at the initial and final visits for patients with congenital hearing loss. There were 10 cases of bilateral severe to profound hearing loss, 6 cases of unilateral severe hearing loss, and 3 cases of unilateral moderate hearing loss, respectively.

### 3.2. Hearing Loss at the Last Visit

Among congenital hearing loss patients, the severity and laterality of hearing loss at the last visit were as follows: bilateral severe to profound hearing loss in 14 cases (four cases progressed from unilateral hearing loss); unilateral severe to profound hearing loss in four cases (two cases progressed from unilateral moderate hearing loss); and severe on one side and moderate on the other side in one case (this case progressed from unilateral moderate hearing loss). Among seven cases with progression, six cases had intellectual disability.

Among later onset hearing loss patients, the severity and laterality of hearing loss at the last visit were as follows: bilateral severe to profound hearing loss in eight cases, unilateral severe to profound hearing loss in three cases, bilateral ski slope hearing loss in one case, and unilateral mild hearing loss in one case. The four cases with unknown onset consisted of three cases of bilateral severe-to-profound hearing loss and one case of unilateral severe-to-profound hearing loss.

Table 2 shows the variety of hearing loss severity and laterality among patients with congenital, later onset, or unknown onset hearing loss. Hearing loss progressed in severity and laterality; 69% (25/36) of patients with congenital, later onset, or unknown onset had bilateral severe-to-profound hearing loss. Among congenital hearing loss patients, 74% (14/19), and among later onset hearing loss patients, 62% (8/13) eventually progressed to severe to profound bilateral hearing loss.

### 3.3. Progression Timing

**Figure 2:**
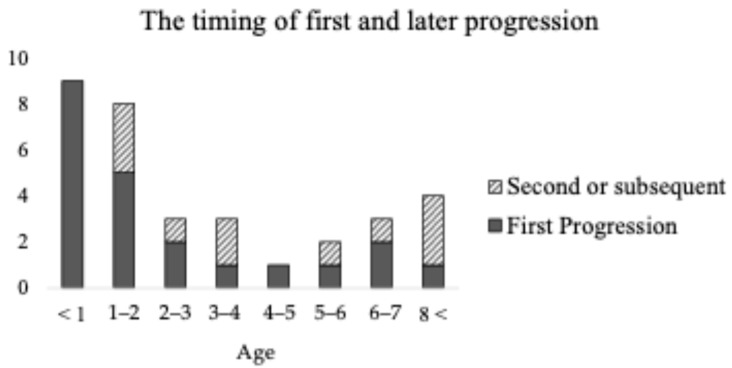
The number of patients of each age at the first progression and second or subsequent progressions. The horizontal axis shows the age at the progression, and the vertical axis shows the number of patients.

Of the 36 patients with congenital, later onset, or unknown onset sensorineural hearing loss, 13 patients experienced later onset hearing loss and 22 patients (61%), including those with congenital hearing loss, experienced progression of their hearing loss. Figure 2 shows the timing of this progression. Fifty percent (11/22) of patients experienced multiple episodes of hearing loss progression. The timing of the first progression was under one year old in nine cases, between one and three years old in seven cases, between three and seven years old in four cases, and after eight years old in one case. The second or subsequent progression occurred between one and three years old in four cases, between three and seven years old in three cases, and after eight years old in three cases, respectively.

### 3.4. Intellectual Disability

Among the 32 patients with congenital or later onset sensorineural hearing loss, 14 had intellectual disability and 18 did not. Table 3 shows the number of cases of congenital and later onset sensorineural hearing loss in patients with and without intellectual disability. Among patients with intellectual disability, 79% (11/14) had congenital sensorineural hearing loss, and 21% had later onset sensorineural hearing loss. Among patients without intellectual disability, 44% (8/18) had congenital sensorineural hearing loss, and 56% (10/18) had later onset sensorineural hearing loss. Patients with intellectual disability tended to have congenital sensorineural hearing loss more often than patients without intellectual disability. However, there was no significant difference between the two groups (*p* = 0.07).

## 4. Discussion

In this study, we evaluated the patterns and long-term progression of hearing loss in patients with congenital CMV infection. We found that 67% of patients (36 out of 54) eventually experienced sensorineural hearing loss. Of the patients with hearing loss, at least 36% (13/36) experienced later onset hearing loss, and 61% (22/36), including those with congenital hearing loss, experienced hearing loss progression. Progression occurred at various times after birth, half of the patients who experienced hearing loss progression had multiple progression events, and 69% (25/36) of the patients with sensorineural hearing loss eventually progressed to bilateral severe to profound sensorineural hearing loss. Patients with intellectual disability tended to show congenital sensorineural hearing loss, but there was no significant difference.

Congenital CMV infection can cause either congenital or later onset hearing loss. Some cases of CMV infection are diagnosed when patients show signs of hearing loss. One main hypothesis is that the stria vascularis is the target organ damaged by CMV. In a mouse model, injecting CMV into the fetus primarily caused stria vascularis dysfunction, leading to loss of endocochlear potential maintenance [7,8]. Another study found that CMV-infected mice showed higher permeability of the blood–cochlear barrier. The researchers hypothesized that CMV infection damages the integrity of the blood–cochlear barrier, disturbing the homeostasis of the inner ear [9]. Immune response is considered another cause of hearing loss [10,11]. CMV infection increases reactive oxygen species, activates nucleotide-binding oligomerization domain-like receptor protein 3 (NLRP3) inflammatory bodies in cochlear and spiral ganglion cells, and activates Caspase 1, leading apoptosis [12]. Although various mechanisms have been proposed to explain CMV-induced hearing loss, no hypothesis can fully account for the variety of hearing loss due to CMV infection, including laterality, severity, and progression. The main factor affecting the hearing threshold remains unknown.

Progression of hearing loss due to congenital CMV infection sometimes occurs. The mechanism causing progression is unclear, but two main hypotheses have been proposed. One hypothesis is that continuous CMV infection directly damages hair cells or spiral ganglion cells. Another hypothesis is that the host immune reaction activated by CMV infection damages infected cells [13]. The presence of CMV DNA in children’s inner ear perilymph suggests that CMV persists in patients’ inner ears and affects them not only during the acquisition period, but also later in life. A recent review study concluded that the rate of later onset hearing loss is between 1.3% and 35% [14]. Other studies have suggested that 20% of cases of unilateral hearing loss progress to bilateral hearing loss and that the risk of progression from unilateral to bilateral hearing loss is high [5,15]. Our study also showed that some patients with CMV infection experienced later onset hearing loss, and that patients with congenital hearing loss progressed.

In this study, we also identified the timing of progression. A recent study suggested that the risk of hearing loss after five years in cCMV infection with isolated hearing loss patients was no higher than in normal children [16]. However, few studies have revealed hearing data followed for more than ten years [15,17], and data on long-term hearing progression with CMV infection is limited. One study showed that the poorer-hearing ear progressed first at an average age of two years, and the better-hearing ear progressed first at an average age of six years [17]. Our study also clarified that progression occurred over more than 5 years and that multiple progressions occurred, suggesting that long-term follow-up is needed for such patients.

We also found that 69% (25/36) of the patients with sensorineural hearing loss eventually progressed to bilateral severe to profound sensorineural hearing loss. Previous studies have suggested that approximately 30% of the patients suffer bilateral severe to profound hearing loss [18,19], which is a smaller percentage than in our study.

One reason our study showed a higher percentage of patients with severe hearing loss is the population we included. We did not include all patients diagnosed with congenital CMV infection. Our institution performs cochlear implant surgeries, and patients with severe to profound bilateral hearing loss tend to be referred to us. This selected group may have a higher potential for severe hearing loss. Another reason is our long-term follow-up period. As mentioned above, most studies have shorter follow-up periods than ours. The long follow-up period revealed the hidden number of patients who eventually suffered severe to profound hearing loss. As hearing loss severity progressed, interventions such as hearing aids or cochlear implants were needed to prevent delayed language development. Especially in children with bilateral severe to profound hearing loss, CI is needed for language development. Therefore, long-term close monitoring of patients’ hearing ability is necessary, even if their hearing severity remains normal or mild.

Although there was no significant difference, patients with intellectual disability tended to have congenital hearing loss more often. A previous study suggests that patients with more severe clinical features due to CMV infection experience a greater amount of viral invasion of the fetus [20]. Another study showed that fewer patients with cCMV infection with isolated hearing loss had detectable CMV DNA in their perilymph [13]. These findings suggest that a larger amount of the virus causes inner ear damage earlier, leading to congenital hearing loss. It should be noted that patients without intellectual disability may still experience hearing loss later in life. A former study revealed that patients with symptomatic cCMV infection showed congenital hearing loss in 32–41% of cases, whereas patients with cCMV infection with isolated hearing loss showed congenital hearing loss in 7–10% of cases [21]. This paper also elucidated that cCMV infection in patients with isolated hearing loss had a higher risk of later onset hearing loss or progression. This corresponds to our findings, suggesting that patients with cCMV infection with isolated hearing loss should pay more attention to their hearing levels.

In our study, we found no evidence that antiviral therapy, which, in a randomized trial, suggested the improvement of hearing outcomes in the long term [22], is effective in preventing hearing loss progression. However, we could not draw conclusions about the effects of antiviral therapy, because our patient group received an inconsistent antiviral therapy strategy, including in factors such as patient selection, treatment age, and treatment duration.

Our study has several limitations. This is a single-site study and therefore not necessarily representative of a wider cCMV population, and it is a retrospective study with all the inherent problems of a non-prospective cohort. In addition, it is not representative of congenital CMV infection overall, because we did research on symptomatic cCMV infection and cCMV infection with isolated hearing loss, and there is another category of asymptomatic cCMV with no HL. This group is actually the largest of the cCMV groups, and are also at risk of later onset HL but were not included. The limited number of patients is also a limitation of our study. This small sample size may not be sufficient to demonstrate the wide variety of clinical presentations in patients with CMV-induced hearing loss. Patients confirmed to have normal ABR findings in pediatrics and for whom no subsequent hearing loss has been suspected have not attended our department. Although regular attendance is recommended, some patients have discontinued their appointments of their own accord. Patients without complaints tend to avoid the hospital, which could increase the rate of hearing loss, especially late-onset hearing loss. However, our study demonstrated the variability in the literature regarding severity and progression of hearing loss, which may have overcome this limitation. Furthermore, it is difficult to determine whether later onset hearing loss in congenital CMV patients is actually caused by CMV infection. However, sudden sensorineural hearing loss rarely occurs during childhood, and many later onset hearing loss cases in patients with congenital CMV were attributed to CMV infection. Moreover, it is difficult to rule out hearing progression before the first detection of hearing loss among later onset hearing loss patients or between the follow-up periods. Not all patients receive audiological assessments regularly, which could delay progression findings. Lastly, our institution is a tertiary care center where we perform cochlear implants, so selection bias could exist. Our patients tend to have more severe hearing loss (10 cases of bilateral severe/profound, 6 cases of unilateral severe/profound, and 3 cases of unilateral moderate) than those of other reports [23,24,25], and the feature of being a third-tier center offering closer follow-up and detailed hearing evaluations leads to a more severe estimation of the level of hearing loss progression.

## 5. Conclusions

Of the patients with CMV-associated hearing loss, 36% (13/36) had later onset hearing loss, and 61% (22/36) experienced progression of hearing loss. Progression occurred in terms of both severity and laterality. Progression mainly occurs before the age of one year, though several cases experienced progression in children over one year old, including some cases after the age of five years. Patients with CMV without intellectual disability tend to experience later onset hearing loss. Fifty percent (11/22) of patients experienced multiple episodes of hearing loss progression. Additionally, 69% (25/36) of patients with congenital, later onset, or unknown onset had bilateral severe to profound hearing loss, necessitating continuous long-term follow-up.

## Figures and Tables

**Table 1:** The demographic of the patients who have received valganciclovir. Patient 10 suffered pancytopenia as a side effect of valganciclovir, and treatment was stopped. F; female, ID; intellectual disability, M; male, mo.; month, mos.; months, wks.; weeks, y.o.; years old, yr.; years, yrs.; years.

No.	Age at First Visit	Onset	Symptomatic	ID	Treatment Age	Treatment Duration	Sex	Hearing at Last Visit	Progression
1	5 mos.	Congenital	Symptomatic	Yes	1 mo.	6 mos.	F	Unilateral profound and unilateral moderate	Yes
2	2 mos.	Later onset	Symptomatic	Yes	3 wks.	6 mos.	F	Unilateral severe	Yes
3	17 y.o.	Unknown	Symptomatic	Yes	6 yrs.	6 mos.	M	Bilateral profound	Yes
4	2 mos.	Congenital	Isolated HL	No	1 mo.	6 mos.	M	Unilateral profound	No
5	6 mos.	Congenital	Symptomatic	Yes	3 wks.	6 mos.	F	Unilateral profound	No
6	4 mos.	Congenital	Symptomatic	Yes	1 mo.	6 mos.	M	Unilateral profound	Yes
7	1 mo.	Later onset	Symptomatic	No	3 mos.	6 mos.	M	Unilateral moderate	Yes
8	1 yr. 3 mos.	Congenital	Symptomatic	Yes	1 wk.	6 mos.	M	Unilateral moderate	Yes
9	5 mos.	Congenital	Symptomatic	Yes	3 wks.	6 mos.	F	Bilateral profound	Yes
10	2 yrs. 3 mos.	Congenital	Symptomatic	Yes	3 wks.	1 mo.	M	Bilateral profound	Yes
11	4 yrs. 0 mo.	Congenital	Isolated HL	No	1 mo.	6 wks	F	Bilateral severe	Yes

**Table 2:** The degree and laterality of hearing loss among congenital, later onset, and unknown onset hearing loss patients.

	Bilateral Severe/Profound (*n*, %)	Other Bilateral (*n*, %)	Unilateral (*n*, %)	Total
Congenital	14 (74)	1 (5)	4 (21)	19
Symptomatic	8 (67)	1 (8)	3 (25)	12
Isolated HL	6 (86)	0 (0)	1 (14)	7
Later onset	8 (62)	1 (8)	4 (31)	13
Symptomatic	2 (40)	1 (20)	2 (40)	5
Isolated HL	6 (75)	0 (0)	2 (25)	8
Unknown onset	3 (75)	0 (0)	1 (25)	4
Symptomatic	2 (100)	0 (0)	0 (0)	2
Isolated HL	1 (50)	0 (0)	1 (50)	2
Total	25 (69)	2 (6)	9 (25)	36
Symptomatic	12 (63)	2 (11)	5 (26)	19
Isolated HL	13 (76)	0 (0)	4 (24)	17

**Table 3:** The number of patients with congenital or later onset hearing loss among patients with intellectual disability and without intellectual disability. ID; intellectual disability.

	With ID (*n*, %)	Without ID (*n*, %)	Total
Congenital hearing loss	11 (79)	3 (21)	14
Later onset hearing loss	8 (44)	10 (56)	18

## Data Availability

Raw data were generated at the authors’ institution. Derived data supporting the findings of this study are available from the corresponding author (K.A.) on request.

## Abbreviations

The following abbreviations are used in this manuscript:

CMV	Cytomegalovirus
ID	Intellectual Disability
